# Draft Genome Sequence of *Bacillus subtilis* Strain S1-4, Which Degrades Feathers Efficiently

**DOI:** 10.1128/genomeA.00766-13

**Published:** 2013-09-26

**Authors:** Bin Yong, Bin-Qing Yang, Chuan-Wu Zhao, Hong Feng

**Affiliations:** Sichuan Key Laboratory of Molecular Biology and Biotechnology, Key Laboratory of Bio-resources and Eco-environment of the Ministry of Education, College of Life Sciences, Sichuan University, Sichuan, People’s Republic of China

## Abstract

*Bacillus subtilis* strain S1-4, with the capacity to efficiently degrade feathers, was isolated from chicken feathers. Sequencing showed that the genome of strain S1-4 differs from that of other *B. subtilis* strains, with limited insertions and deletions. The genome encodes multiple extracellular proteases and keratinases.

## GENOME ANNOUNCEMENT

Feathers from the poultry industry are a valuable protein source and can be transformed into soluble peptides and free amino acids (1, 2). Many microorganisms have been isolated and demonstrated to be able to degrade feathers. Keratinase and disulfite reductase activities are recognized as the main mechanisms for degrading keratin (3–9). *Bacillus subtilis* strain S1-4, which was isolated from chicken feathers from a local poultry farm in China, degrades chicken feathers efficiently. It can secrete several extracellular proteases which hydrolyze various substrates (keratin, casein, and gelatin) (10). In order to gain insight regarding the genetic information encoding the keratinolytic enzymes in *B. subtilis* S1-4, we sequenced the genome of *B. subtilis* S1-4 using the Illumina HiSeq 2000 at the Shenzhen Huada Genomics Institute (China).

The sequencing generated about 1.0 Gb paired-end reads with lengths of 90 bp, which covered the whole genome about 200 times. The clean reads were assembled using ABySS software (11). We obtained 103 contigs with an *N*_50_ of 43,179 bp, and the largest contig was 280,067 bp. The draft genome sequence contains a total of 4,447,441 bp with a G+C content of 43.1%, about 0.23 Mb more than that of the model strain *B. subtilis* 168. In addition, a circular plasmid with a length of 6,609 bp was assembled. The genome phylogenetic trees inferred from the whole orthologs and constructed using PhyML (12) showed that S1-4 was closely related to (in order from most to least closely related) *B. subtilis* strains (168, SMY, and JH642, etc.), *B. amyloliquefaciens* strain FZB52, and *B. licheniformis* strain ATCC 14580.

The draft genome sequence was annotated with the RAST system (13). A total of 114 RNA-encoding genes and 4,935 coding sequences (CDS) were identified, among which 3,499 CDS were assigned to 1 of the 589 RAST subsystems. The numbers of predicted genes involved in the metabolism of DNA, nitrogen, protein, carbohydrates, phosphorous, and potassium did not show obvious difference from those for *B. subtilis* 168. In contrast, the CDS in the S1-4 genome sequence belonging to the subsystems of membrane transport, sulfur metabolism, mobility and chemotaxis, regulation, and cell signaling were much more numerous than those in strain 168. However, the putative genes encoding extracellular proteases and peptidases did not show any significant differences between strains S1-4 and 168, except that some single-nucleotide polymorphisms (SNPs) occurred between these genes. In addition, some CDS encoding putative reductases were present in S1-4. This genome sequence information indicated that the keratinolytic activities of strain S1-4 may be derived from proteases and putative reductases. Using Mauve software (14), we carried out comparative genome analysis with the closest strains of *Bacillus*. Overall, the sequences of S1-4 and 168 were collinear, with only limited insertions and deletions. Several regions containing a putative prophage, a type II restriction-modification system, and mobile elements, which may be obtained from horizontal gene transfer in natural niches, were present in the S1-4 genome sequence.

### Nucleotide sequence accession number.

The sequence of *Bacillus subtilis* S1-4 under this whole-genome shotgun project has been deposited at DDBJ/EMBL/GenBank under accession number ANIP00000000.
